# Stage-Specific Tumoral Gene Expression Profiles of Black and White Patients with Colon Cancer

**DOI:** 10.1245/s10434-024-16550-9

**Published:** 2024-11-23

**Authors:** Mohamad El Moheb, Chengli Shen, Susan Kim, Kristin Putman, Hongji Zhang, Samantha M. Ruff, Russell Witt, Allan Tsung

**Affiliations:** 1 School of Data Science, University of Virginia, Charlottesville, VA USA; 2 Department of Surgery, University of Virginia, Charlottesville, VA USA

## Abstract

**Background:**

Black patients with colon cancer (CC) exhibit more aggressive tumor biology and higher treatment resistance than white patients, even after adjusting for clinical and demographic factors. We investigated stage-specific transcriptional differences in tumor profiles of Black and white patients with CC.

**Patients and Methods:**

Patients with CC from The Cancer Genome Atlas Colon Adenocarcinoma database were categorized by disease stage and propensity-score matched between Black and white patients. Differential gene expression and pathway enrichment analyses were performed for each stage. Logistic regression and quadratic discriminant analysis (QDA) models were developed using consistently differentially expressed genes.

**Results:**

Of 247 patients, 128 had localized (22% Black), 81 had regional (74% Black), and 38 had distant disease (29% Black). Differential expression analysis revealed differences in 312 genes for localized, 105 for regional, and 199 for distant stages between Black and white patients. Pathway enrichment analysis showed downregulation of the IL-17 pathway in Black patients with localized disease. In total, five genes exhibited race-specific transcriptional differences across all stages: *RAMACL, POLR2J3, POLR2J2, MUC16,* and *PRSS21.* Logistic regression and QDA model performance indicated that these genes represent racial differences [area under the receiver operating characteristic curve (AUC): 0.863 and 0.880].

**Conclusions:**

Significant transcriptional differences exist in CC between Black and white patients changing dynamically across disease stages, and involving genes with broad functions. Key findings include IL-17 pathway downregulation in Black patients with localized disease and a five-gene signature consistent across all stages. These findings may explain aspects of racial disparities in CC, emphasizing the need for race-specific research and treatment strategies.

**Supplementary Information:**

The online version contains supplementary material available at 10.1245/s10434-024-16550-9.

Colon cancer is the third leading cause of cancer incidence and mortality in the USA.^1^ While the overall mortality has steadily decreased owing to improved screening practices, early intervention, and advancements in treatment,^1^ it is not evenly distributed across races.^2^ Even after accounting for socioeconomic status,^3^ access to care, and treatment modalities, Black patients have consistently worse survival outcomes compared with white patients at every disease stage.^4–7^ The aggressive disease course in Black patients is often attributed to higher stage at presentation, greater nodal disease burden, and a higher proportion of proximal cancers, suggesting potential underlying differences in tumor biology.^8^

While prior studies explored the putative biologic mechanisms of disease, such as differences in mutational burden and epigenetic profiles between Black and white patients,^9,10^ it is not well understood whether these molecular-level differences directly translate to observed tumor behavior. To address this link between molecular changes and clinical phenotype, efforts are focused on examining the differences in tumor gene expression profiles between these patient populations. However, the existing body of evidence is limited by examining only isolated biologic pathways,^11–13^ small sample sizes,^14^ and lack of stage-specific data.^14,15^ Identifying the exact gene expression differences for each stage could provide important insights into the mechanisms of disease progression and treatment resistance. Cancer research and treatment development are predominantly on the basis of data from white patients,^16,17^ leaving a gap in our understanding of the gene expression profile of Black patients. This disparity limits the effectiveness of current therapies for Black patients, as these treatments are developed from sequencing information that does not represent their unique genetic profiles. By elucidating these profiles, more effective, tailored therapies can be developed to bridge the survival gap between Black and white patients. Furthermore, this may give patients and investigators greater confidence in anticipating treatment response thereby increasing the enrollment of Black patients in randomized controlled trials and ensuring more inclusive and representative clinical research.

This study sought to comprehensively identify stage-specific differences in gene expression profiles between Black and white patients to identify racially specific gene expression profiles associated with outcome disparities.

## Patients and Methods

This study was deemed exempt from the University of Virginia institutional review board (IRB) approval owing to the publicly available and deidentified nature of the dataset.

### Data Source

**Fig. 1:**
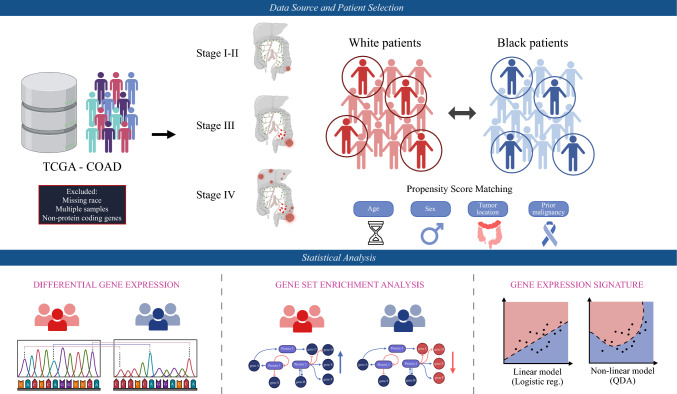
Study schema

The study schematic is illustrated in Fig. 1. Patients with histologically confirmed diagnosis of colon adenocarcinoma included in The Cancer Genome Atlas Colon Adenocarcinoma (TCGA-COAD) database were analyzed. TCGA is publicly available through the National Cancer Institute Genomic Data Commons.^18^ Patients with missing race data and multiple samples were excluded. Patients were categorized into one of three groups depending on disease stage: localized (stage I–II), regional (stage III), and metastatic (stage IV). Baseline characteristics of Black and white patients, including sex, age, tumor location, and prior malignancy, were compared for each disease group, and 4:1 nearest neighbor propensity score matching was used to address imbalances. Race was self-identified by patients.

### Differential Gene Expression and Gene-Set Enrichment Analysis

RNA sequencing data were analyzed to identify differentially expressed genes (DEGs) in the primary tumor samples of Black and white patients for each disease stage. The RNA sequencing methods are previously described.^19^ DEGs were defined by a cutoff of normalized log2-fold changes (log2FC) ≥ 1.0 for overexpression and < −1.0 for under-expression, with an adjusted *p*-value < 0.05 to correct for multiple comparisons using the Benjamini–Hochberg method.

Partial least square (PLS) analysis was performed for all differentially expressed genes to determine the separation between the gene expression profiles of Black patients and white patients. PLS is a supervised machine-learning methodology that reduces the number of dimensions, mitigating the risk of overfitting owing to the large number of genes relative to the number of patients. It also avoids multicollinearity from the co-expression of certain genes and identifies the most significant components that differentiate the groups. The number of PLS components was set to five.

Given the large number of DEGs identified (*n* = 561), this study focused on the 20 most differentially expressed genes in each stage group (i.e., with the highest/lowest log2FC value) to objectively assess those with the highest potential impact related to racial disparities. The broader impact of all DEGs was examined by conducting a gene-set enrichment analysis (GSEA) to identify biologic pathways that are enriched versus suppressed in Black patients. The functional and pathway enrichment knowledge of the DEGs were provided by Kyoto Encyclopedia of Genes and Genomes (KEGG; available online: http://www.kegg.jp/) database. Differential gene expression analysis and GSEA were conducted using the DESeq2 and Clusterprofiler packages in R.^20,21^

To explore potential relationships between the most significant genes identified in this analysis, a pairwise correlation analysis was performed using Pearson’s correlation coefficient. This analysis focused on the 20 genes showing the highest differential expression across disease stages in Black patients compared with white patients. Examining these correlations can reveal potential gene–gene interactions and their roles in disease mechanisms. Strongly correlated genes may be part of the same pathway, regulate each other's expression, or be coregulated by common factors.

### Transcriptomic Signature

Genes consistently differentially expressed across all stages were analyzed to identify a unique transcriptomic signature for Black patients. Two classifiers were constructed using tenfold cross-validation to determine how well these genes can differentiate between Black and white patients: a logistic regression model (a linear model) and a quadratic discriminant analysis (QDA) model (a non-linear model) to capture potential complex nonlinear interactions in gene expression. The performance of these models, measuring the ability of these genes to distinguish between Black and white patients, was assessed using accuracy, precision, and the area under the receiver operating characteristic curve (AUC).

### Survival Analysis

Kaplan–Meier curves were constructed to estimate overall survival (OS) and progression-free survival (PFS) between Black and white patients. Hypothesis testing using the log-rank test was performed to assess statistical significance in OS and PFS between Black and white patients. Survival analysis first compared white versus Black patients across all disease stages, followed by a stage-specific breakdown.

Given the limited number of patients in the TCGA dataset, which may not fully represent survival trends across the broader US population, we utilized the NCDB database to conduct a nationwide survival analysis. This allowed us to corroborate disparities in survival between white and Black patients with colon cancer, as previously reported in literature.^5–7^ Patients were stratified into three groups based on disease stage, and a multivariable Cox proportional hazards regression was performed for each group to compare OS between white and Black patients. The analysis adjusted for sociodemographic factors (age, sex, insurance status), comorbidities (Charlson–Deyo Comorbidity Class), cancer characteristics (laterality, facility type, time from diagnosis to treatment initiation), treatment modalities (surgery, chemotherapy, immunotherapy), and year of diagnosis. Results were presented as hazard ratios (HR) with 95% confidence intervals (CI).

The threshold for statistical significance was set at *p* < 0.05. All analyses were conducted using R software, version 4.3.1.

## Results

### Patient Characteristics

A total of 247 patients were included in the study. The mean age was 64.6 [standard deviation (SD): 13.4] years, and 51% were male. Among the patients, 128 had localized disease (104 white and 24 Black), 81 had regional disease (60 white and 21 Black), and 38 had metastatic disease (27 white and 11 Black). Data were balanced between groups, except for age in patients with localized disease [white: 68.4 (SD: 12.1) versus Black: 62.5 (SD: 15.0), *p* = 0.044]. Therefore, 96 white patients were propensity score matched to 24 Black patients on the basis of all characteristics. No differences in characteristics were observed after matching (Table 1).

**Table 1:** Characteristics of study population after propensity score matching

	Localized			Regional				Metastatic			
	White (*n* = 96)	Black (*n* = 24)	*p*	White (*n* = 60)		Black (*n* = 21)	*p*	White (*n* = 27)		Black (*n* = 11)	*p*
Age, mean (SD), years	67.5 (12.2)	62.5 (15)	0.09	62.3 (13.8)		61.9 (12.7)	0.83	61.9 (12.9)		56.7 (14.6)	0.29
Male	53 (55%)	9 (38%)	0.19	30 (50%)		11 (52%)	1	13 (48%)		8 (73%)	0.31
Tumor location			0.56								
Left colon	33 (34%)	5 (21%)	0.43	21 (35%)		5 (24%)	0.53	13 (48%)		4 (36%)	0.74
Right colon	48 (50%)	15 (62%)		26 (43%)		12 (57%)		11 (41%)		6 (55%)	
Unspecified	15 (16%)	4 (17)		13 (22%)		4 (19%)		3 (11%)		1 (9%)	
Previous malignancy	7 (7%)	0 (0%)	0.38	6 (10%)		1 (5%)	0.78	4 (14%)		0 (0%)	0.44
Systemic therapy											
Yes	9 (11%)	5 (22%)	0.36	35 (71%)		18 (86%)	0.33	18 (72%)		10 (91%)	0.41
No	70 (89%)	18 (78%)		14 (29%)		3 (14%)		7 (28%)		1 (9%)	

### Partial Least Square Analysis Differential Gene Expression

**Fig. 2:**
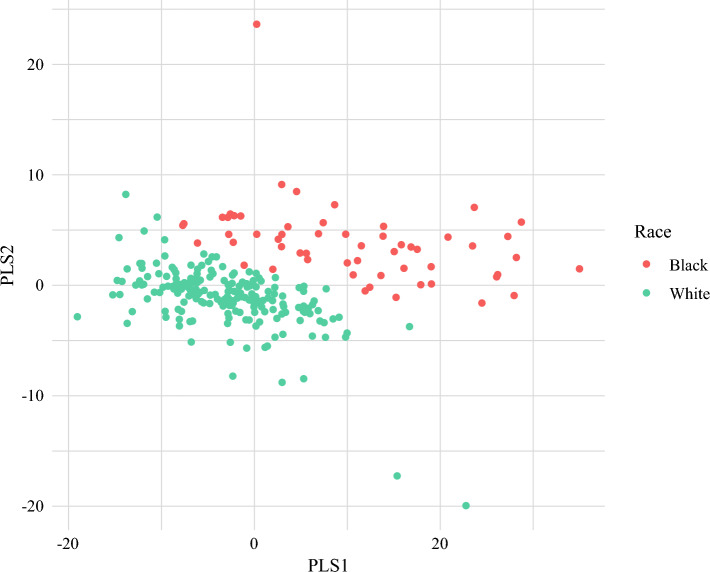
Partial least square (PLS) projection of differentially expressed genes between colon cancer tumor samples from Black and white patients. Projection demonstrates clear separation in the tumor gene expression profile of Black and white patients, suggesting significant underlying molecular differences between the two groups

**Fig. 3:**
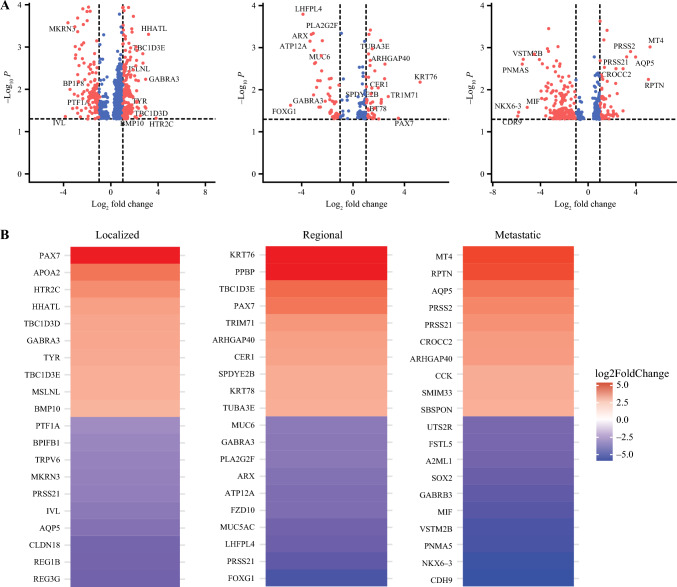
Differential gene expression analysis of Black versus white Patients with colon cancer. **A** Volcano plots of differentially expressed genes between colon cancer tumor samples of Black versus white patients for each disease stage. **B** Heatmaps of top 20 differentially expressed genes between colon cancer tumor samples from Black versus white patients for each disease stage

Partial least square analysis of gene expression data showed clear separation in the tumor samples of Black and white patients (AUC: 0.975), indicating high degree of clustering in the gene expression profile between the two groups (Fig. 2). A total of 561 genes were differentially expressed between Black and white patients, confirming the results of the PLS analysis that there exist gene expression differences between the two groups. Figure 3 presents the Volcano Plot of all the differentially expressed genes, as well as the heatmap showing the 20 most differentially expressed genes between Black and white patients for each disease group. The Log2 Fold change and adjusted *p*-values are provided in Supplementary Table 1. The DEGs can be broadly categorized into seven functional groups in how they relate to cancer: angiogenic and tumor migration (e.g., PRSS2, PRSS21, BP1FB1, MT4-MMP, AQP5, SOX2), immune response and inflammation (e.g., MIF, MSLN, PPBP), metabolic processes (e.g., APOA2, CCK), signal transduction and cellular communication (e., GABRA3, HTR2C, HHATL, TBC1D3D/TBC1D3E), cellular adhesion and structure (e.g., CDH9, KRT78, KRT76, MUC5AC, MUC6), transcription regulation and development (e.g., PTF1A, PAX7, NKX6-3), and other functions (e.g., MKRN3, PNMA5, FSTL5). The function of each of the genes is described in Table 2. The function of the remaining genes has not been described in cancer.

**Table 2:** Classification and function of highest differentially expressed genes

Gene	Stage	Expression in Black patients	Function
Angiogenic and tumor migration			
PRSS21	Localized Regional Metastatic	Under Under Over	Inhibits ovarian tumor metastasis by antagonizing proangiogenic proteins^22^
BP1FB1	Localized	Under	Enhances the activity of FGFs by releasing them from the ECM. FGFs are essential for tumor progression by promoting angiogenesis, cell proliferation, and survival.
REG1B	Localized	Under	Involved in cell proliferation, differentiation, and regeneration. Overexpression associated with more aggressive tumor behavior, and downregulation leads to G1-phase cell cycle arrest^41^
PRSS2	Metastatic	Over	Inhibits the expression of thrombospondin-1 in the tumor microenvironment by binding to low-density lipoprotein receptor-related protein 1. This promotes tumor growth and progression by modifying the tumor microenvironment to favor cancer cell proliferation and metastasis.^42^ Potential target for pharmaceutical intervention with Fisetin^43^
MT4-MMP	Metastatic	Over	Involved in degrading the extracellular matrix, facilitating tumor invasion and metastasis. Enhances EGFR activation and signaling, promoting tumor cell proliferation. Modulates interactions between tumor and stromal components, affecting angiogenesis and immune cell recruitment, which support tumor growth and metastasis^24,25^
AQP5	Metastatic	Over	Induces cell proliferation through activation of the Ras signaling pathway. Involved in the EMT process, crucial for cancer metastasis. Silencing AQP5 in colorectal cancer cells impairs their migration and invasion capabilities. Contributes to multidrug resistance in CC^44^
SOX2	Metastatic	Under	Plays a critical role in cancer metastasis by facilitating EMT, enhancing cell migration and invasion through the WNT and Rho-ROCK signaling pathways.^45^ Promotes angiogenesis and vasculogenic mimicry.^46^ It maintains cancer stem cell properties, contributing to tumor initiation, chemoresistance, and aggressiveness via the β-catenin and Beclin1/autophagy pathways^47^
SBSPON	Metastatic	Over	Mediates metastasis-associated in colon cancer 1 (MACC1) gene-induced colorectal cell proliferation, invasion, and metastasis^23^
Immune response and inflammation			
MIF	Metastatic	Under	Modulates the immune response by increasing the generation of regulatory T cells, which suppress antitumor immunity and allows the tumor to grow unchecked.^48^ Helps cancer cells survive under hypoxic conditions, contributing to resistance against therapies like chemotherapy and radiation^49^
MSLN	Localized	Over	Expression correlates with elevated inhibitory immune activity, increased M1/M2 macrophage infiltration, PD-L1 staining, and immune-inhibitory gene expression in patients with CC. Associated with chemoresistance^33^
PPBP	Regional	Over	Associated with angiogenesis and poor prognosis in CC. Stimulates tumor cell proliferation and enhances aerobic glycolysis. High levels are linked to shorter disease-free and overall survival in CC with liver metastases^50,51^
Metabolic processes			
APOA2	Localized	Over	Promotes tumorigenesis and progression by enhancing cell proliferation and survival, and protecting against apoptosis.^52^ Overexpression linked to resistance to chemoradiation in rectal cancer^53^
CCK	Metastatic	Over	Overexpression linked with poor prognosis and lower disease-free survival.^54^ Anti-CCK2R agents demonstrate high specificity and potential as therapeutic targets^55^
Signal transduction and cellular communication			
GABRA3	Localized	Over	Promotes tumor cell proliferation and survival through alterations in intracellular signaling pathways that support cell growth and division. GABAergic signaling can be targeted for treatment, with Nembutal and GABAAR antagonists, such as flumazenil and bicuculline^36,56,57^
HTR2C	Localized	Over	Associated with cell proliferation and the calcium signaling pathway. Promotes CC progression and carcinogenesis through RhoA/ROCK/YAP signaling, NLRP3 inflammasome activation, and angiogenic signals.^39^ Inhibition with Mirtazapine reduces tumor growth and prolonged survival in mice^38,58^
HHATL	Localized	Over	Catalyzes the Shh signaling pathway, controlling tumorigenesis in a variety of cancers, and is a powerful prognostic gene for patients with ccRCC^53,59,60^
TBC1D3D/TBC1D3E	Localized	Over	Inhibits EGFR degradation, enhancing its signaling pathway and promoting proliferation.^61^ High levels are associated with poor prognosis and immune infiltration in various cancers^62^
Cellular adhesion and structure			
CDH9	Metastatic	Under	Downregulated in CC and may play a role in epithelial-to-mesenchymal transition. Suppressor of tumor metastasis^63,64^
KRT78	Regional	Over	Overexpressed in CC and could be a potential biomarker^65^
KRT76	Regional	Over	Modulates immune responses in patients with head and neck cancer. High expression linked to worse survival in CC.
MUC5AC	Regional	Under	Enhances cell invasion, migration, and decreases apoptosis in CC cells by interacting with CD44 and activating Src signaling, promoting tumorigenesis and metastasis. It also confers resistance to chemotherapy by downregulating p53 and p21 and upregulating β-catenin and its target genes^66^
MUC6	Regional	Under	Overexpression is associated with better PFS and cancer-free survival, particularly in stage II–III disease. White patients with intermediate stage disease exhibited higher expression of these genes, correlating with better survival outcomes^67^
Transcription regulation and development			
PTF1A	Localized	Under	Protects against pancreatic cancer by acting as a tumor suppressor and keeping acinar cells in their healthy, differentiated state^68^
PAX7	Localized, Regional	Over	Responsible for cancer cachexia. Driven by NF-κB signaling. Overexpression prevents progenitor cells from differentiating into mature muscle fibers, impairing muscle regeneration and contributing to muscle atrophy. Potential therapeutic target to prevent muscle wasting in cancer cachexia^26,69^
NKX6-3	Metastatic	Under	Belongs to the NKX family of homeodomain-containing transcription factors, which are involved in tissue-specific differentiation and development and are associated with various cancers. Prevents EMT, cell migration and metastasis^70^
Other functional proteins			
MKRN3	Localized	Under	Tumor suppressor gene through regulating ubiquitination of PABcP1. Low expression associated with poor survival in non-small cell lung cancer^71^
PNMA5	Metastatic	Under	Increases cellular proliferation, invasion and migration in CC Higher expression levels correlate with worse outcomes^72^
FSTL5	Metastatic	Under	Inhibits invasion through the Wnt/β-catenin/YAP pathway in hepatocellular carcinoma^73^

*CC* colon cancer, *ECM* extracellular matrix, *EGFR* epidermal growth factor receptor, *EMT* epithelial–mesenchymal transition, *FGF* fibroblast growth factors

For localized disease, there were 312 DEGs, of which 55% were overexpressed in Black patients compared with white patients. For regional disease, there were 105 DEGs, of which 54% were overexpressed in Black patients. For metastatic disease, there were 199 DEGs, of which 29% were overexpressed in Black patients. Notably, PRSS21, which was under-expressed in Black patients with localized or regional disease, was overexpressed in Black patients with metastatic disease (Supplementary Table 1).

### Gene-Set Enrichment Analysis

**Fig. 4:**
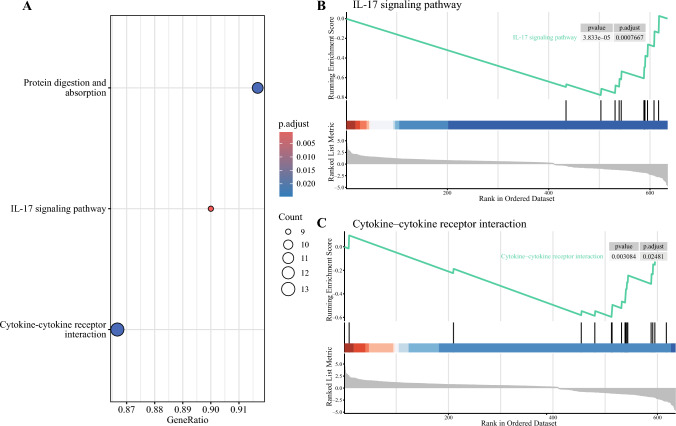
Gene Set Enrichment Analysis (GSEA) of patients with localized colon cancer. **A** Dotplot of GSEA results. **B** Suppression of the IL-17 signaling pathway in Black patients compared with white patients. **C** Suppression of the cytokine-cytokine receptor interaction pathway in Black patients compared with white patients

Gene set enrichment analysis was performed to evaluate the potential functional relevance of DEGs and determine if certain biologic pathways are upregulated or downregulated in Black patients compared with white patients. Black patients with localized disease exhibited suppression of the IL-17 signaling pathway (Fig. 4), evidenced by the under-expression of key pro-inflammatory cytokines and chemokines, including CXCL5, CXCL6, CXCL8, CXCL10, GM-CSF, MUC5AC, and IFN gamma compared with white patients. Additionally, Black patients with localized disease showed suppression of the cytokine–cytokine receptor interaction pathway owing to the under-expression of the abovementioned cytokines, along with other immune modulators, such as IL-11, IL-24, and CXCL11. No enriched pathways were identified in patients with regional or metastatic disease.

### Transcriptional Signature

**Fig. 5:**
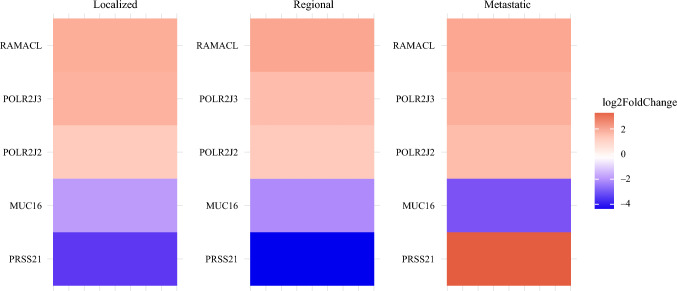
Heatmap of the five commonly differentially expressed genes across disease stages between colon cancer tumor samples from Black versus white patients

This study then sought to identify genes that are commonly differentially expressed between Black and white patients across all stages to uncover consistent molecular markers that could potentially serve as universal indicators of racial differences in colon cancer. In total, five genes were consistently differentially expressed across all disease stages: *RAMACL, POLR2J3, POLR2J2, MUC16,* and *PRSS21*. RAMACL, POLR2J3, POLR2J2, and MUC16 gene expression was consistent across all disease stages, either consistently over or under-expressed in Black patients. However, the expression of PRSS21 shifted from being highly under-expressed in localized and regional disease to overexpressed in metastatic disease (Fig. 5). The function of this gene has not yet been described in the context of colon cancer, but it is a known inhibitor of primary ovarian tumor metastasis by antagonizing pro-angiogenic protein.^22^

To evaluate if these five genes represent the gene expression profiles of Black patients, we built logistic regression and quadratic discriminant analysis (QDA) models. The logistic regression model achieved 0.883 accuracy, 0.895 precision, and 0.863 AUC. The QDA model achieved 0.796 accuracy, 0.925 precision, and 0.880 AUC. These results suggest these five genes could serve as universal indicators of the unique gene expression profile of Black patients, regardless of disease stage.

### Gene Co-Expression Analysis

Given that PRSS21 was the most differentially expressed gene regardless of disease stage, a pairwise correlation analysis identified genes correlated with PRSS21 expression. In regional disease, PRSS21 is significantly correlated with FOXG1. In metastatic disease, PRSS21 overexpression correlated with MT4-MMP and SBSPON, both involved in tumor invasion and metastasis.^23–25^ These findings suggest a possible interaction between PRSS21 and key genes promoting tumor invasiveness in Black patients with colon cancer (Supplementary Fig. 1).

A pairwise correlation analysis of all other genes identified potential gene–gene interactions. Significant co-upregulation of APOA2 and PAX7 was found in Black patients with localized disease, both linked to cachexia. PAX7 prevents progenitor cells from becoming mature muscle fibers, leading to muscle atrophy.^26^ APOA2, involved in the acute-phase response and inflammation, modulates serum amyloid A levels, promoting cachexia.^27,28^

### Survival Analysis

Survival analysis compared overall survival (OS) and progression-free survival (PFS) between Black and white patients (Supplementary Figs. 2 and 3). No significant OS differences were found overall (median: 61.8 months versus 92.7 months, *p* = 0.55) or by disease stage: localized (Black: not reached, white: 92.7 months, *p* = 0.15), regional (Black: 56.2 months, white: not reached, *p* = 0.40), metastatic (Black: 61.8 months, white: 28.2 months, *p* = 0.71). Black patients had lower PFS overall (55.1 months versus 84.2 months, *p* = 0.039). Significant PFS differences were found for regional disease (*p* = 0.044) but not for localized (59.2 months versus not reached, *p* = 0.22) or metastatic disease (35.1 months versus 21.7 months, *p* = 0.83).

Given the small sample size and potential for selection bias, we conducted a survival analysis comparing OS between Black and white patients using NCDB data. A total of 323,711 patients were analyzed (Supplementary Fig. 4), of which 29% had localized disease, 33% had regional disease, and 38% metastatic disease. The characteristics of the population are summarized in Supplementary Table 2. Black patients with colon cancer had significantly worse survival rate at every disease stage compared with white patients [localized: HR (95% CI) = 1.11 (1.09; 1.14), regional: HR = 1.06 (1.02; 1.11), metastatic: HR = 1.07 (1.04; 1.11), Supplementary Tables 3–5].

### Sensitivity Analysis

A sensitivity analysis was performed on patients with localized (stage I–II) and regional (stage III) disease to identify genes that consistently show differential expression between the two groups, excluding the potential influence of systemic therapy in stage IV disease. This analysis revealed 26 such genes (Supplementary Fig. 5), with all but one maintaining consistent correlation patterns. Notably, 26% of DEGs in regional disease were also differentially expressed in localized disease, indicating a higher number of consistent DEGs than the initially noted five and suggesting more stable gene expression patterns between closer disease stages.

## Discussion

Significant efforts have been directed toward understanding racial disparities in cancer treatment outcomes. Even when clinical and demographic variables are matched, Black patients with CC consistently experience worse outcomes at every disease stage compared with white patients.^3,4^ This was recently highlighted in a study by Yousef et al. that demonstrated worse outcomes among Black patients, including a poorer response to first-line chemotherapy compared with white patients. The authors identified genetic mutations as the second most significant factor driving these disparities, suggesting differences in tumor phenotype that may be reflected through gene expression changes—a topic that remains underexplored.^29^ As such, this study performed an in-depth investigation of the tumor gene expression profiles of these two groups to uncover the underlying biologic processes that contribute to observed disparities in clinical outcomes.

This study revealed significant transcriptional differences between Black and white patients with colon cancer. PLS analysis of gene expression data revealed distinct clustering patterns, with Black and white patients separated by their tumor transcription profiles. Differential gene expression analysis identified 561 DEGs, further confirming these molecular differences. A key strength of this study is its comprehensive examination of DEGs across different colon cancer stages, addressing limitations in previous research that focused on specific pathways or had a limited scope of genetic profile differences.^11–15^ This approach is crucial, as disease resistance and progression mechanisms vary with cancer stage.^30^ These results showed that gene expression differences were not uniform across stages. This study identified 312 DEGs in localized disease, 105 in regional disease, and 199 in metastatic disease when comparing Black versus white patients. This stage-specific variation in DEGs suggests that molecular differences in tumors between Black and white patients are dynamic, evolving with disease progression and potentially reflecting changes in tumor behavior. Surprisingly, metastatic disease, which is typically more heterogeneous, exhibited fewer DEGs compared with localized disease, which is generally considered less heterogeneous.

Given the large number of genes in each stage, a systematic approach was necessary to objectively assess the functions of those with the highest potential for explaining differences in outcomes. Therefore, this study analyzed the 20 highest differentially expressed genes in each stage. Among the angiogenic genes, PRSS21, a serine protease, was under-expressed in Black patients with localized or regional disease. Although its role in CC is unexplored, PRSS21 is known to inhibit primary ovarian tumor metastasis by antagonizing proangiogenic proteins.^22^ Interestingly, PRSS21 was under-expressed in Black patients with localized or regional disease but overexpressed in those with metastatic disease. The significance of this is unclear, but it could represent a change in expression that promotes metastatic disease, influence from the tumor microenvironment at metastatic sites resulting in overexpression of PRSS21, or suggest that overexpression of PRSS21 correlates to more aggressive biology and a higher propensity for metastatic disease. Specifically, we found that PRSS21 overexpression in Black patients with metastatic disease was co-upregulated with two other genes, MT4-MMP and SBSPON, both associated with aggressive tumor behavior.^23–25^ This suggests that having a gene expression profile with upregulation of these genes may predict high tumor invasiveness in Black patients.

Another significant finding of this study is the altered tumor immune microenvironment in Black patients, potentially contributing to immune evasion and disease progression. Both TBC1D3 and MSLN have immunosuppressive functions and are overexpressed in Black patients with localized disease. TBC1D3, shares high homology with other TBC domain genes that contribute to colorectal cancer carcinogenesis through M2 macrophage infiltration, creating an immunosuppressive tumor microenvironment that facilitates immune evasion and tumor progression.^31,32^ Similarly, mesothelin (MSLN) is associated with immune inhibition in CC, allowing cancer cells to proliferate undetected.^33^ Furthermore, the pathway enrichment analysis demonstrated that Black patients with localized disease had downregulation of the IL-17 and cytokine-cytokine receptor interaction pathways. Suppression of IL-17 signaling leads to increased recruitment of CD8+ cytotoxic T lymphocytes (CTLs) to colorectal tumors through upregulation of chemokines CXCL9 and CXCL10.^34^ IL-17 suppression also results in increased infiltration of regulatory T cells (Tregs) in the tumor microenvironment. While Tregs generally suppress anti-tumor immune responses, their increased presence alongside CTLs suggests a complex interplay that may involve balancing pro- and anti-inflammatory signals. Blocking IL-17 signaling increases the expression of anti-inflammatory cytokines, such as IL-10 and TGF-β. These cytokines can modulate the immune response and potentially create a more immunosuppressive microenvironment, which might counteract some of the benefits of increased CTL infiltration.^34^ Interestingly, IL-17 suppression is shown to improve the efficacy of immune checkpoint inhibitors (ICIs), such as anti-PD-1 therapy, in microsatellite stable colorectal cancer by reducing IL-17-mediated pro-tumorigenic inflammation and enhancing CTL activity within the tumor microenvironment.^35^ This suggests that Black patients might respond better to ICIs than white patients; however, further research is needed to test this hypothesis.

The importance of this analysis extends beyond understanding biologic differences; it has potential implications for developing targeted therapies. Historically, most genetic and transcriptomic research that forms the basis for ongoing efforts in cancer drug research and development predominantly relied on data from White patients.^16,17^ This bias potentially limits the efficacy of treatments for diverse populations. Our analysis addresses this gap by identifying several DEGs with potential therapeutic implications in Black patients. Notably, neurotransmitter-related genes, such as GABRA3 and HTR2C, present promising targets. GABRA3 (GABA type A receptor subunit alpha3) was significantly overexpressed in Black patients with localized disease, and GABA receptor antagonists, such as flumazenil, exhibit antiproliferative effects.^36,37^ Similarly, the activity of HTR2C (serotonin 2C receptor), which is upregulated in Black patients, could be inhibited with Mirtazapine, which showed reduced tumor growth and prolonged survival in in vivo studies.^38,39^ PAX7 is another promising therapeutic target. This gene, linked to muscle development and cancer-related cachexia,^26^ was significantly overexpressed in Black patients with localized and regional disease, with levels 30 folds higher than white patients. Additionally, PAX7 was co-expressed with APOA2 in patients with localized disease, which modulates important acute phase response proteins, such as serum amyloid A, involved in the pathogenesis of cachexia.^27,28^ These findings support clinical studies and provide a molecular rationale that could explain why Black patients with gastrointestinal cancer are much more likely to develop cachexia than white patients.^40^ While direct PAX7 inhibitors are not yet available, recent research suggests that reducing PAX7 expression or modulating its downstream targets could reverse muscle wasting associated with cachexia.^40^

To identify a unique tumoral gene expression signature specific to Black patients with colorectal cancer, this study examined genes that are differentially expressed across all stages. This analysis revealed five such genes: RAMACL, POLR2J3, POLR2J2, MUC16, and PRSS21. Among these, PRSS21 showed the highest expression levels in Black patients, further supporting its potential role as a key molecular marker for tumors in this population. Two predictive models, logistic regression and QDA, were built to assess these genes’ representation of racial differences in tumoral gene expression. Both models demonstrated high AUC, precision, and accuracy in classifying patients by race on the basis of these five genes. This approach, which examines how gene expression profiles define racial differences rather than how race influences gene expression, provides strong evidence that these five genes effectively capture the racial differences in tumoral gene expression profiles, and underscores the importance of continued research into genetic differences in diverse populations to develop more effective targeted therapies.

The findings of this study provide critical insights into the genetic differences contributing to racial disparities in CC outcomes. Our research is the first to comprehensively assess gene expression differences between Black and white patients with CC in a stage-specific manner, overcoming significant limitations of previous studies.^11–15^ The identification of key differentially expressed genes and unique transcriptional signatures provides potential pathways for future research and therapeutic targeting. However, our study has limitations inherent to gene expression analysis from a cohort derived from the TCGA. First, while we found that PFS was worse for Black patients compared with white patients, there was no difference in OS between the two groups, despite evidence in literature to the contrary.^5^ The lack of OS difference in our study is likely attributable to our relatively low sample size, limiting the power of our analysis. However, our cohort could also represent a select group of patients that outperform the real-world population. The patients analyzed may have had better access to care or more comprehensive follow-up, mitigating the typically observed survival disparities. In fact, Black patients in the TCGA data were at least as likely to receive treatment as white patients. This finding may not be representative of the broader US population, where Black patients often face barriers to accessing care, indicating potential selection bias in our sample. As such, validation in larger, more diverse cohorts is needed. Second, we did not have any information regarding systemic therapy received, which could impact gene expression levels. Third, the analysis was based on data from RNA sequencing of tumor samples, which does not account for tumor heterogeneity. Additionally, because this is based on bulk transcriptomic sequencing there may be sequencing data from the nearby tumor microenvironment and not just the tumor cells included in the analysis. Future studies with spatial transcriptomics that can separate the cancer cells from neighboring epithelial cells may provide more specific information.

## Conclusions

This study reveals important disparities in the gene expression profiles of Black versus white patients with CC. These differences are stage-specific and dynamic, evolving with progressing disease stages, and encompass a broad range of biologic functions. Notably, downregulation of the IL-17 pathway in Black patients suggests a modulated tumor immune microenvironment more conducive to early-stage tumor progression. We have also identified a unique gene expression signature specific to Black patients, with PRSS21 emerging as having a potential role in CC. Our findings highlight the need for race-specific cancer research and treatment. Understanding unique genetic profiles can lead to targeted, effective therapies, improving prognosis and survival rates for Black patients with colon cancer in precision medicine.

## Supplementary Information

Below is the link to the electronic supplementary material.

Supplementary file1 (DOCX 1031 KB)
