# Supplementary material for: Stage-Specific Tumoral Gene Expression Profiles of Black and White Patients with Colon Cancer
Source: Ann Surg Oncol. 2024 Nov 23;32(2):736–49. doi: 10.1245/s10434-024-16550-9 (PMC11698818; doi:10.1245/s10434-024-16550-9)
Supplement: Supplementary file 1 — Supplementary file1 (DOCX 1031 KB) [file 10434_2024_16550_MOESM1_ESM.docx]

**SUPPLEMENTARY MATERIAL**

**Table 1. Top 20 differentially expressed genes of colon cancer tumor samples of Black and White patients with localized, regional, and metastatic disease. Reference group is White patients.**

|  | **Localized** | | | **Regional** | | | **Metastatic** | | |
| --- | --- | --- | --- | --- | --- | --- | --- | --- | --- |
|  | **Gene** | **Log2FC** | **Adj. P** | **Gene** | **Log2FC** | **Adj. P** | **Gene** | **Log2FC** | **Adj. P** |
| **OVEREXPRESSED IN BLACK PATIENTS** | PAX7 | 6.59 | 1.68e-10 | KRT76 | 5.22 | 0.006 | MT4 | 5.22 | 9.38E-04 |
|  | APOA2 | 4.53 | 1.34e- 7 | PPBP | 5.22 | 6.78E-11 | RPTN | 5.08 | 0.006 |
|  | HTR2C | 3.81 | 0.048 | TBC1D3E | 3.82 | 4.20e- 5 | AQP5 | 4.01 | 0.002 |
|  | HHATL | 3.17 | 5.01e- 4 | PAX7 | 3.53 | 0.046 | PRSS2 | 3.59 | 0.001 |
|  | TBC1D3D | 2.98 | 0.028 | TRIM71 | 2.74 | 0.015 | PRSS21 | 3.26 | 0.002 |
|  | GABRA3 | 2.93 | 0.006 | ARHGAP40 | 2.5 | 0.0024 | CROCC2 | 2.97 | 0.003 |
|  | TYR | 2.9 | 0.026 | CER1 | 2.44 | 0.0055 | ARHGAP40 | 2.93 | 0.032 |
|  | TBC1D3E | 2.71 | 0.001 | SPDYE2B | 2.22 | 0.017 | CCK | 2.47 | 0.041 |
|  | MSLNL | 2.71 | 0.002 | KRT78 | 2.2 | 0.021 | SMIM33 | 2.46 | 0.012 |
|  | BMP10 | 2.5 | 0.041 | TUBA3E | 2.14 | 6.78e- 4 | SBSPON | 2.38 | 0.038 |
| **UNDER-EXPRESSED IN BLACK PATIENTS** | PTF1A | -3.24 | 0.028 | MUC6 | -3.03 | 0.0015 | UTS2R | -3.9 | 0.016 |
|  | BPIFB1 | -3.44 | 0.010 | GABRA3 | -3.06 | 0.0133 | FSTL5 | -3.92 | 0.011 |
|  | TRPV6 | -3.57 | 1.46e- 5 | PLA2G2F | -3.07 | 4.53e- 4 | A2ML1 | -4.07 | 0.002 |
|  | MKRN3 | -3.63 | 2.68e- 4 | ARX | -3.23 | 4.78e- 4 | SOX2 | -4.47 | 0.001 |
|  | PRSS21 | -3.68 | 5.22e- 6 | ATP12A | -3.33 | 7.00e- 4 | GABRB3 | -4.82 | 1.11E-06 |
|  | IVL | -3.85 | 0.041 | FZD10 | -3.33 | 1.56e- 7 | MIF | -5.09 | 0.026 |
|  | AQP5 | -4.05 | 5.90e- 9 | MUC5AC | -3.69 | 5.42e- 5 | VSTM2B | -5.43 | 0.002 |
|  | CLDN18 | -4.49 | 2.18e- 7 | LHFPL4 | -3.89 | 1.60e- 4 | PNMA5 | -5.52 | 0.002 |
|  | REG1B | -4.55 | 4.97e- 8 | PRSS21 | -4.33 | 1.18e- 6 | NKX6-3 | -5.82 | 0.034 |
|  | REG3G | -4.71 | 4.69e- 5 | FOXG1 | -4.85 | 0.023 | CDH9 | -5.88 | 0.042 |

Abbreviations: Log2FC: Log_2_ Fold Change; Adj. P: adjusted p-value

**Table 2. Characteristics of National Cancer Database Patient Population Analyzed for Surival Analysis**

| **Characteristics** | **White patients (N = 275,760)** | **Black patients  (N = 47,951)** | **p-value** |
| --- | --- | --- | --- |
| Age, years, median [IQR] | 70 (59, 79) | 64 (54, 74) | <0.001 |
| Sex, female | 139,102 (50%) | 25,474 (53%) | <0.001 |
| Insruance |  |  | <0.001 |
| None | 6,795 (2.5%) | 3,281 (7.0%) |  |
| Private | 91,184 (34%) | 15,871 (34%) |  |
| Medicare | 160,346 (59%) | 21,615 (46%) |  |
| Medicaid | 10,849 (4.0%) | 5,559 (12%) |  |
| Other | 2,275 (0.8%) | 485 (1.0%) |  |
| Charlson-Deyo Score |  |  | <0.001 |
| 0 | 192,111 (70%) | 33,203 (69%) |  |
| 1 | 57,144 (21%) | 10,202 (21%) |  |
| 2 | 17,494 (6.3%) | 2,853 (5.9%) |  |
| 3 | 9,011 (3.3%) | 1,693 (3.5%) |  |
| Facility type |  |  | <0.001 |
| Community cancer program | 29,266 (11%) | 3,364 (7.3%) |  |
| Comprehensive Community Cancer Program | 119,056 (44%) | 15,158 (33%) |  |
| Academic/Research Program | 68,145 (25%) | 18,731 (40%) |  |
| Integrated Network Cancer Program | 52,296 (19%) | 8,998 (19%) |  |
| Stage |  |  | <0.001 |
| Localized | 138,331 (50%) | 19,790 (42%) |  |
| Regional | 36,199 (13%) | 6,204 (13%) |  |
| Metastatic | 101,230 (37%) | 21,957 (46%) |  |
| Laterality |  |  | <0.001 |
| Left-side | 96,487 (38%) | 16,819 (39%) |  |
| Right-side | 159,144 (62%) | 26,663 (61%) |  |
| Chemotherapy | 110,871 (41%) | 21,661 (47%) | <0.001 |
| Immunotherapy | 12,686 (4.6%) | 2,767 (5.8%) | <0.001 |
| Surgery |  |  | <0.001 |
| None | 58,337 (21%) | 13,909 (29%) |  |
| Localized | 76,156 (28%) | 10,725 (22%) |  |
| Partial colectomy | 127,982 (46%) | 20,971 (44%) |  |
| Total colectomy | 8,672 (3.1%) | 1,419 (3.0%) |  |
| Unspecified surgery | 4,268 (1.5%) | 865 (1.8%) |  |
| Time from diagnosis to treatment, days, median [IQR] | 9 (0, 26) | 9 (0, 30) | <0.001 |

**Table 3. Multivariable Cox Regression Model Comparing Overall Survival Between Black and White Patients with Localized Disease**

| **Variables** | **Hazard Ratio [95% CI]** |
| --- | --- |
| Age | 1.06 [1.06 ,1.06] |
| Female sex | 0.81 [0.79 ,0.82] |
| Black *(ref: White)* | **1.11 [1.09 ,1.14]** |
| Insurance *(ref: None)* |  |
| Private | 0.58 [0.55 ,0.62] |
| Medicare | 0.68 [0.64 ,0.72] |
| Medicaid | 1.08 [1 ,1.17] |
| Other | 0.67 [0.6 ,0.76] |
| Charlson-Deyo Score *(ref: None)* |  |
| 1 | 1.31 [1.29 ,1.34] |
| 2 | 1.78 [1.73 ,1.83] |
| 3 | 2.27 [2.19 ,2.35] |
| Facility type *(ref: Community cancer program)* |  |
| Comprehensive Community Cancer Program | 0.99 [0.96 ,1.01] |
| Academic/Research Program | 0.91 [0.89 ,0.94] |
| Integrated Network Cancer Program | 0.97 [0.94 ,1] |
| Laterality *(ref: Left side)* |  |
| Right side | 0.96 [0.94 ,0.98] |
| Surgery *(ref: None)* |  |
| Local excision | 0.19 [0.18 ,0.2] |
| Partial colectomy | 0.2 [0.18 ,0.21] |
| Total colectomy | 0.26 [0.24 ,0.28] |
| Unspecified surgery | 0.22 [0.19 ,0.24] |
| Time from diagnosis to treatment, days | 0.99 [0.99, 0.99] |
| Year of Diagnosis | 1.00 [0.99, 1.01] |

**Table 4. Multivariable Cox Regression Model Comparing Overall Survival Between Black and White Patients with Regional Disease**

| **Variables** | **Hazard Ratio [95% CI]** |
| --- | --- |
| Age | 1.03 [1.03,1.04] |
| Female sex | 0.84 [0.82,0.86] |
| Black *(ref: White)* | 1.06 [1.02,1.11] |
| Insurance *(ref: None)* |  |
| Private | 0.69 [0.63,0.76] |
| Medicare | 0.81 [0.74,0.89] |
| Medicaid | 1.06 [0.95,1.18] |
| Other | 0.7 [0.58,0.85] |
| Charlson-Deyo Score *(ref: None)* |  |
| 1 | 1.18 [1.14,1.22] |
| 2 | 1.5 [1.43,1.58] |
| 3 | 1.63 [1.52,1.75] |
| Facility type *(ref: Community cancer program)* |  |
| Comprehensive Community Cancer Program | 0.97 [0.93,1.01] |
| Academic/Research Program | 0.91 [0.87,0.96] |
| Integrated Network Cancer Program | 0.97 [0.92,1.02] |
| Laterality *(ref: Left side)* |  |
| Right side | 1.05 [1.02,1.09] |
| Chemotherapy | 0.58 [0.57,0.6] |
| Immunotherapy | 2.09 [1.84,2.38] |
| Surgery *(ref: None)* |  |
| Local excision | 0.22 [0.2,0.24] |
| Partial colectomy | 0.24 [0.21,0.26] |
| Total colectomy | 0.3 [0.26,0.34] |
| Unspecified surgery | 0.24 [0.21,0.28] |
| Time from diagnosis to treatment, days |  |
| Year of Diagnosis | 0.98 [0.98,0.98] |

**Table 5. Multivariable Cox Regression Model Comparing Overall Survival Between Black and White Patients with Metastatic Disease**

| **Variables** | **Hazard Ratio [95% CI]** |
| --- | --- |
| Age | 1.01 [1.01 ,1.02] |
| Female sex | 0.96 [0.94 ,0.97] |
| Black *(ref: White)* | 1.07 [1.04 ,1.09] |
| Insurance *(ref: None)* |  |
| Private | 0.81 [0.77 ,0.84] |
| Medicare | 0.89 [0.85 ,0.93] |
| Medicaid | 0.97 [0.93 ,1.02] |
| Other | 0.85 [0.78 ,0.92] |
| Charlson-Deyo Score *(ref: None)* |  |
| 1 | 1.09 [1.07 ,1.11] |
| 2 | 1.22 [1.18 ,1.27] |
| 3 | 1.51 [1.44 ,1.58] |
| Facility type *(ref: Community cancer program)* |  |
| Comprehensive Community Cancer Program | 0.99 [0.96 ,1.01] |
| Academic/Research Program | 0.81 [0.78 ,0.83] |
| Integrated Network Cancer Program | 0.95 [0.92 ,0.98] |
| Laterality *(ref: Left side)* | 1.17 [1.15 ,1.19] |
| Right side | 1.17 [1.15 ,1.19] |
| Chemotherapy | 0.48 [0.47 ,0.49] |
| Immunotherapy | 0.91 [0.89 ,0.93] |
| Surgery *(ref: None)* |  |
| Local excision | 0.45 [0.44 ,0.46] |
| Partial colectomy | 0.46 [0.45 ,0.47] |
| Total colectomy | 0.43 [0.41 ,0.45] |
| Unspecified surgery | 0.38 [0.35 ,0.4] |
| Time from diagnosis to treatment, days | 0.99 [0.99 ,0.99] |
| Year of Diagnosis | 0.97 [0.97 ,0.98] |


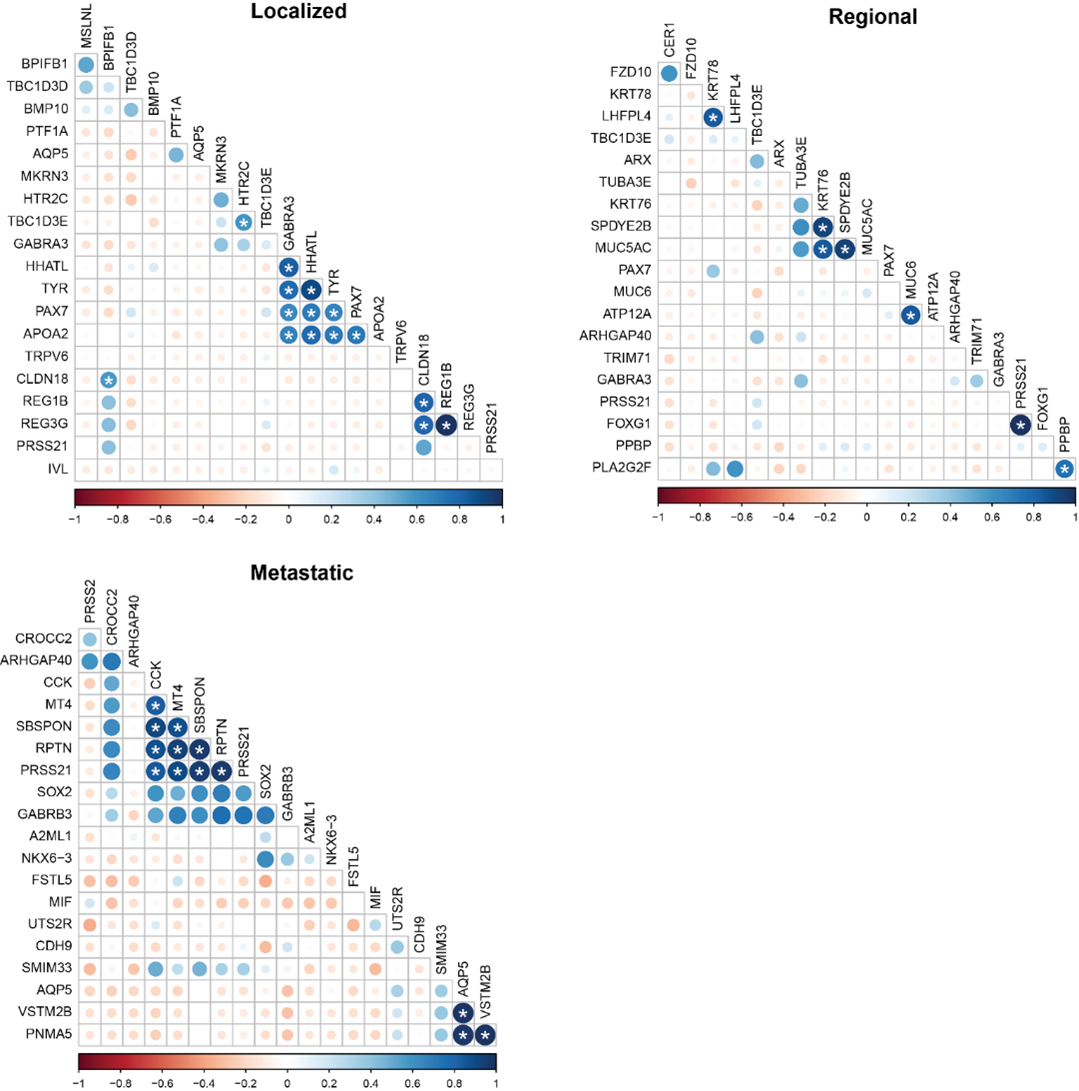


**Supplementary Figure 1. Pairwise correlation of gene co-expression among the 20 highest differentially expressed genes in Black patients across localized, regional, and metastatic disease stages**. The heatmaps display the correlation coefficients, where blue indicates positive correlations and red indicates negative correlations. The size and color of the circles represent the strength of the correlation, and significant correlations (p < 0.05) are marked with asterisks.


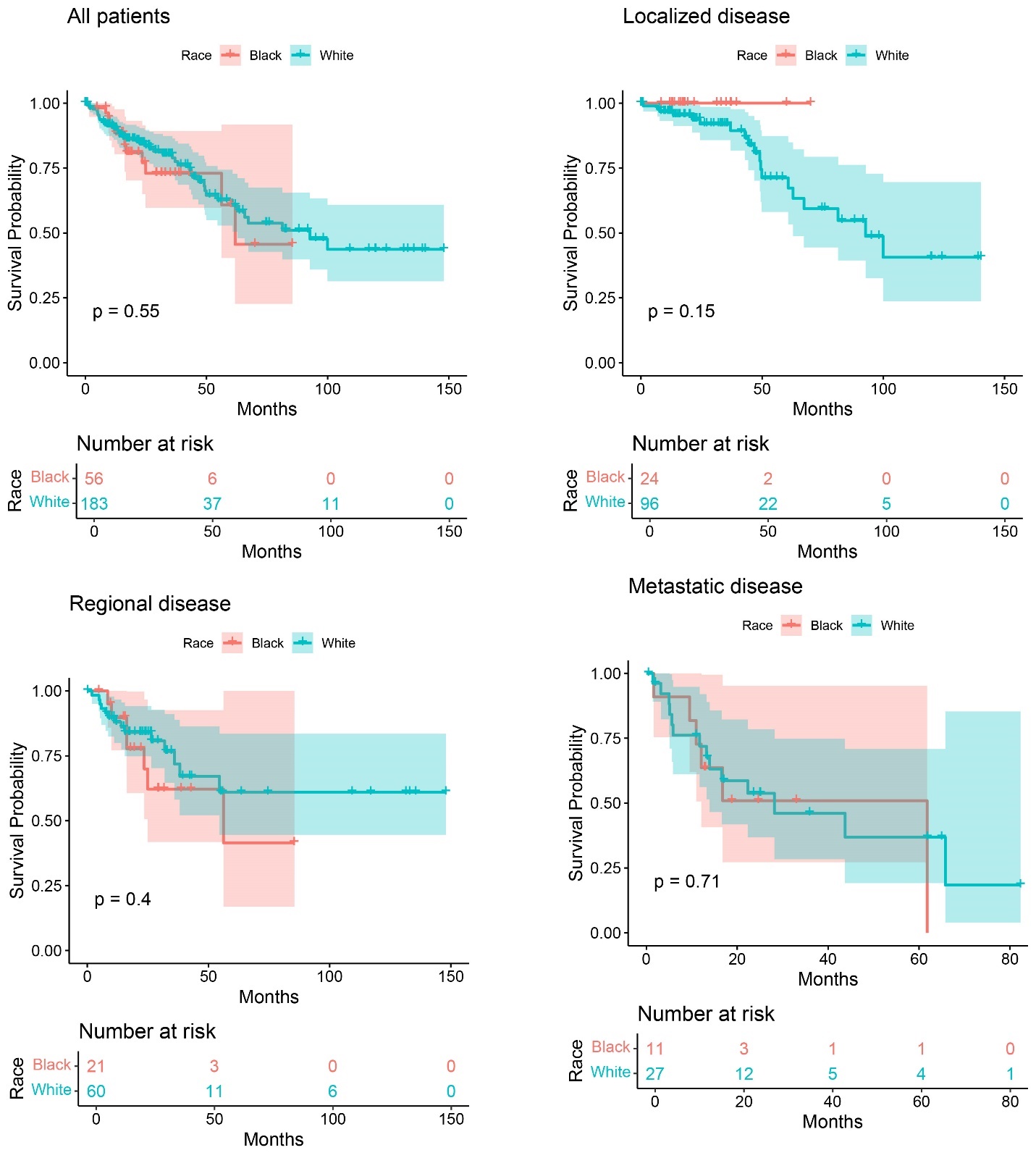


**Supplementary Figure 2. Overall survival by race, for all patients, and then stratified by disease stage.**


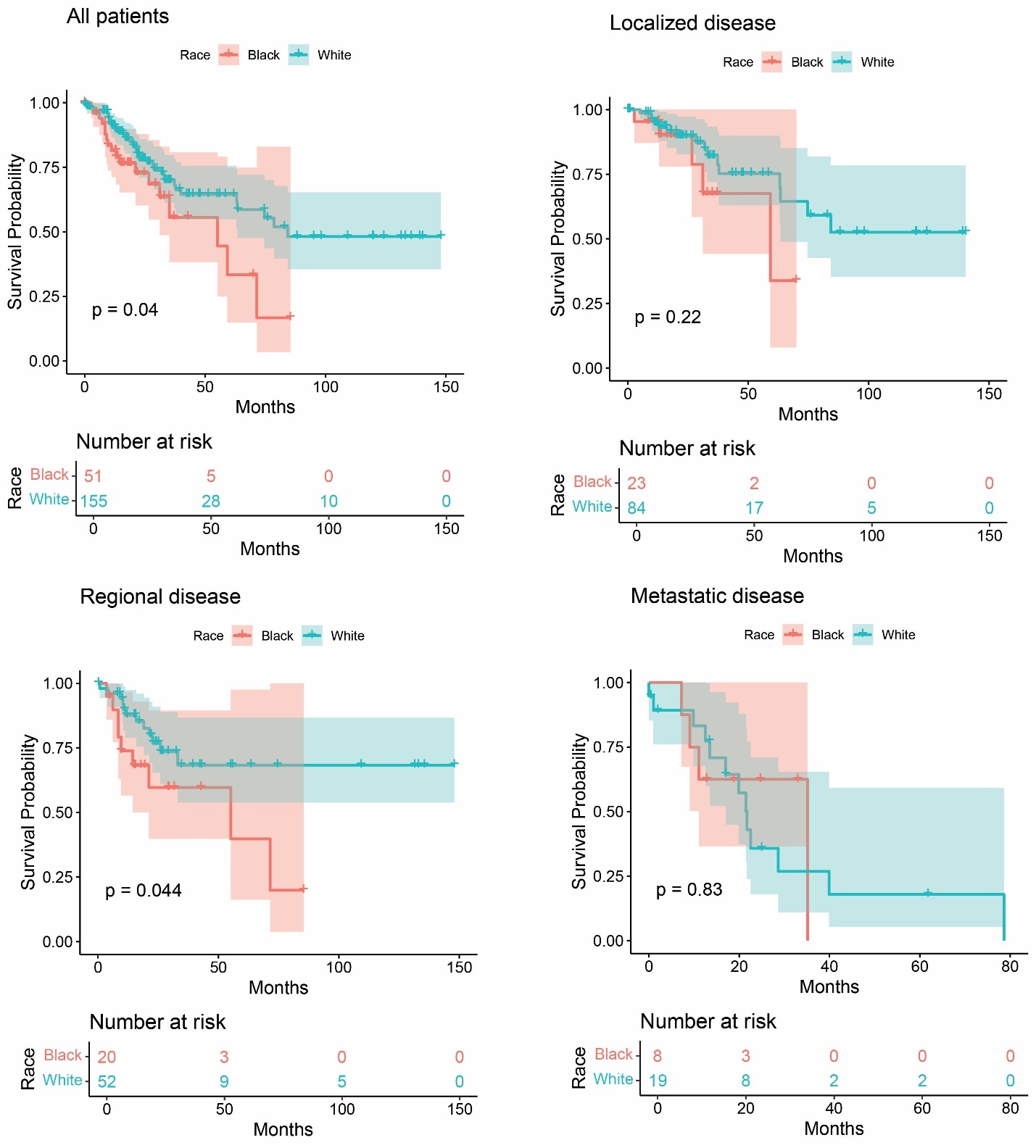


**Supplementary Figure 3. Progression-free survival by race, for all patients, and then stratified by disease stage.**


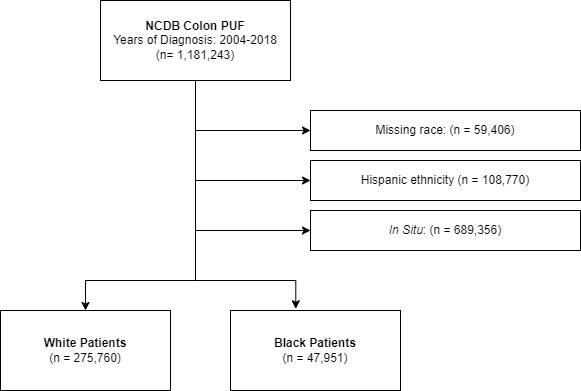


**Supplementary Figure 4. Patient Selection Flowchart for Survival Analysis Using NCDB Colon Cancer Data (2004-2018).**


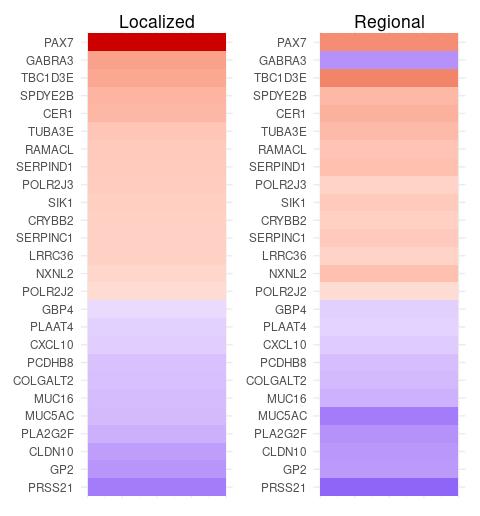
 **Supplementary Figure 5. Heatmap of the 26 commonly differentially expressed genes between localized and regional colon cancer tumor samples of Black versus White patients.**
